# Camrelizumab-Related Myocarditis and Myositis With Myasthenia Gravis: A Case Report and Literature Review

**DOI:** 10.3389/fonc.2021.778185

**Published:** 2022-01-03

**Authors:** Jing Bai, Dan Li, Peidan Yang, Kunyan Xu, Yingnan Wang, Qian Li, Jiang Liu, Wenli Du, Fengbin Zhang, Rui Feng

**Affiliations:** ^1^ Department of Pharmacy, Fourth Hospital of Hebei Medical University, Shijiazhuang, China; ^2^ Department of Gastroenterology, Fourth Hospital of Hebei Medical University, Shijiazhuang, China; ^3^ Department of Thoracic Surgery, Fourth Hospital of Hebei Medical University, Shijiazhuang, China

**Keywords:** camrelizumab, myocarditis, myositis, myasthenia gravis, immune-related adverse events

## Abstract

Immune checkpoint inhibitors (ICIs) have substantially changed the treatment of a variety of malignant tumors. With the increasing of their usage, the unique immune-mediated toxicity profile of ICIs has become apparent. We report a case of esophageal squamous cell carcinoma in a patient who received anti-programmed cell death protein 1 (PD-1) (camrelizumab) therapy and the occurrence of sequential immune-related adverse events (irAEs). Although many irAEs have been reported, severe myositis caused by camrelizumab with simultaneous involvement of multiple organs, including the myocardium, respiratory muscles, and skeletal muscles, has rarely been described in literature. This 69-year-old male patient developed a grade 4 camrelizumab-induced adverse reaction according to the Common Terminology Criteria for Adverse Events (CTCAE) and was successfully treated with methylprednisolone and immunoglobulins. The early identification of irAEs, immediate discontinuation of immunotherapy, use of steroids and/or immunosuppressants, and adjuvant supportive treatment are critical to the clinical prognosis of patients. It should be aware that autoimmune complications can occur even when ICI treatment is ceased.

## Introduction

Malignant tumors have become a major public health problem worldwide (1). Surgery, chemotherapy, and radiotherapy are the main treatment methods used to fight cancers, but the mortality rate from tumors remains very high. Recently, immune checkpoint blockade therapy as immunotherapy has become a modality of cancer treatment. Immune checkpoint inhibitors (ICIs) that target and block the programmed cell death protein 1 pathway (PD-1/PD-L1) and cytotoxic T lymphocyte-associated antigen 4 (CTLA-4) have demonstrated treatment potential in a variety of malignant tumors (2). Since the FDA approved the first CTLA-4 inhibitor, ipilimumab, in 2011 and the first PD-1 inhibitor, pembrolizumab, for melanoma treatment in 2014, ICIs have been approved for the treatment of various other tumors and have shown considerable advantages and impressive results in the field of tumor immunotherapy.

PD-1 is a negative regulator of T cell activity; it can limit the activity of T cells at various stages of the immune response when it interacts with its two ligands, PD-L1 and PD-L, negatively regulates T cell activation, and plays a key role in tumor evasion of host immunity (3). PD-1 inhibitors can block this interaction, which normally leads to T cell activation. However, PD-1 inhibitors induce many immune-related adverse events (irAEs) while activating T cells (4). These irAEs can involve multiple systems and locations, including the gastrointestinal tract, endocrine glands, skin, and liver (5); furthermore, irAEs such as myocarditis, heart failure, rhabdomyolysis, myositis, and myasthenia gravis (MG) have high fatality rates, thus warranting high vigilance in clinical practice (6–9).

Camrelizumab is a PD-1 inhibitor developed by Jiangsu Hengrui Medicine Co. Ltd., China. It was conditionally approved in China in May 2019 for the treatment of relapsed or refractory classic type II Hodgkin’s lymphoma in patients who had received at least second-line chemotherapy (10). Due to its good antitumor potential in a variety of malignant tumors, this drug has been approved for four indications in China to date: advanced esophageal cancer, advanced hepatocellular carcinoma, non-small cell lung cancer, and advanced esophageal squamous cell carcinoma. A number of clinical trials involving multiple indications for camrelizumab are ongoing (**Table 1**).

**Table 1:** Key ongoing clinical trials of camrelizumab in cancers.

Trial Name	Indication	Phase	Status	NCT
Study of SHR-1210 in Combination with Chemotherapy in Advanced Esophageal Cancer	Advanced esophageal cancer	III	Recruiting	NCT03691090
A Trial of SHR-1210 (an Anti-PD-1 Inhibitor) in Combination with FOLFOX4 in Subjects with Advanced HCC Who Have Never Received Prior Systemic Treatment	Advanced hepatocellular carcinoma	III	Recruiting	NCT03605706
Phase III Study of Camrelizumab in Combination with Chemotherapy in Recurrent/Metastatic Nasopharyngeal Carcinoma	Nasopharyngeal carcinoma	III	Active, not recruiting	NCT03707509
A Study to Evaluate SHR-1210 in Subjects with Advanced HCC	Hepatocellular carcinoma (nonresectable)	II	Active, not recruiting	NCT02989922
PD-1 Antibody SHR-1210 in Patients with Relapsed or Refractory Classic Hodgkin’s Lymphoma	Hodgkin lymphoma	II	Recruiting	NCT03155425
A Study to Evaluate SHR-1210 in Patients with Advanced or Metastatic NSCLC	Lung neoplasms, non-small cell lung carcinoma, respiratory tract neoplasms nec, bronchial neoplasm, bronchogenic carcinoma	II	Recruiting	NCT03085069
A Trial of SHR-1210 (an Anti-PD-1 Inhibitor) in Combination with Hypofraction Radiotherapy in Patients With NSCLC	Non-small cell lung cancer	II	Recruiting	NCT03557411
SHR-1210 in Recurrent/Metastatic Nasopharyngeal Carcinoma Who Have Received Previous at Least Two Lines of Chemotherapy	Nasopharyngeal carcinoma	II	Active, not recruiting	NCT03558191
A Study of SHR-1210 in Combination with Apatinib or Chemotherapy in Subjects with Advanced PLC or BTC	Advanced primary liver cancer, advanced biliary tract carcinoma	II	Recruiting	NCT03092895
A Study of SHR-1210 in Combination with Apatinib in Advanced Non-Small Cell Lung Cancer (NSCLC)	Non-small cell lung carcinoma	II	Recruiting	NCT03083041
Neoadjuvant Anti-PD-1 Antibody SHR-1210 and Radiation in Resectable Esophageal Squamous Cell Carcinoma	Esophageal neoplasms	II	Recruiting	NCT03200691
A Trial of SHR-1210 (an Anti-PD-1 Inhibitor) in Combination with Apatinib in Patients with Advanced HCC (RESCUE)	Hepatocellular carcinoma	II	Active, not recruiting	NCT03463876
Famitinib Plus Anti-PD1 Therapy for Advanced Urinary System Tumors, Advanced Gynecological Tumors	Renal cell carcinoma, urothelial carcinoma, cervical cancer, recurrent ovarian cancer, endometrial cancer	II	Recruiting	NCT03827837
SHR-1210 in Combination with GEMOX in Patients with Advanced BTC	Biliary tract cancer, cholangiocarcinoma	II	Recruiting	NCT03486678
A Study of SHR-1210 in Combination with Capecitabine + Oxaliplatin or Apatinib in Treatment of Advanced Gastric Cancer	Gastric cancer, gastroesophageal cancer	II	Active, not recruiting	NCT03472365
The Phase II Trial of SHR-1210 Combined with Preoperative Chemotherapy for Locally Advanced Esophageal Squamous Cell Carcinoma	Locally advanced esophageal squamous cell carcinoma	II	Recruiting	NCT03917966
Neoadjuvant Chemoradiation Plus PD-1 Antibody (SHR-1210) in Locally Advanced Proximal Stomach Adenocarcinoma	Gastric adenocarcinoma	II	Recruiting	NCT03631615
SHR-1210 Combined with Apatinib in the Treatment of ED-SCLC after Failure of First-Line Standard Therapy	Small cell lung cancer	II	Pending	NCT03417895
A Trial of SHR-1210 (an Anti-PD-1 Inhibitor) in Combination with Apatinib and Fluzoparib in Patients with TNBC	Triple negative breast cancer	I	Recruiting	NCT03945604
A Study to Evaluate the Safety and Efficacy of SHR-1210, Gemcitabine and Cisplatin by R/M NPC Subjects	Nasopharyngeal carcinoma	I	Active, not recruiting	NCT03121716

Data from nine clinical trials (n = 986) showed that most camrelizumab recipients experienced an adverse event, 24% of which were grade 3 or higher (10). Adverse events of any severity with an incidence of ≥ 10% included reactive cutaneous capillary endothelial proliferation (RCCEP), anemia, fever, hypothyroidism, fatigue, and proteinuria. RCCEP appears to be unique to patients treated with camrelizumab; moreover, studies have shown that RCCEP is generally a grade 1 or 2 adverse event, and the clinical efficacy of camrelizumab treatment is closely correlated with a survival benefit (11). For grade 3 or grade 4 treatment-related adverse events (TRAEs), common symptoms include elevated aspartate aminotransferase (AST) (12), a decreased neutrophil count (12), pulmonary infection (13), elevated blood bilirubin (12), a decreased platelet count (12), and elevated serum alkaline phosphatase (10). Here, we report a case of MG and myositis involving the myocardium and respiratory muscles induced by the treatment of advanced esophageal cancer with the PD-1 inhibitor camrelizumab. The conditions of the patient improved after treatment.

## Case Presentation

**Figure 1:**
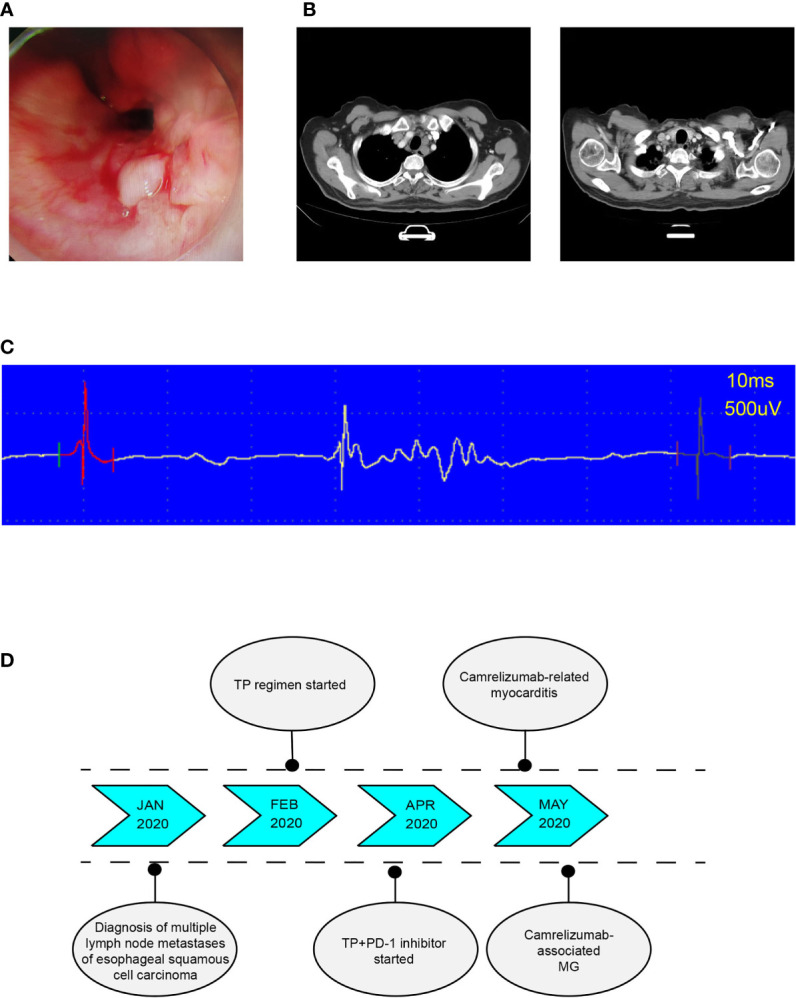
**(A)** Endoscope images showing an ulcerative neoplasia. **(B)** In January 2020, posttreatment CT-scan showed middle and upper esophageal carcinoma with bilateral tracheoesophageal sulcus and right supraclavicular lymph node metastasis. **(C)** Typical electromyogram (EMG) of deltoid muscle showing the patient's myogenic damage. **(D)** Timeline of diagnosis, treatment, and immune-related adverse events.

A 69-year-old male was admitted to the hospital for examination due to hoarseness in January 2020. Gastroscopy showed an ulcerative neoplasia approximately 23 cm-31 cm from the incisors (**Figure 1A**), which resulted in luminal stenosis. Pathological diagnosis revealed poorly differentiated squamous cell carcinoma. Initial cervical-thoracic enhanced computed tomography (CT) showed middle and upper thoracic esophageal carcinoma, suspicious invasion of the fibrous membrane surface, and multiple swollen lymph nodes in the bilateral supraclavicular region, bilateral tracheoesophageal sulcus, and mediastinum (**Figure 1B**). The diagnosis was multiple lymph node metastases of esophageal squamous cell carcinoma. The patient had a history of diabetes for eight years. The patient had normal heart function and therefore received a normal TP regimen (paclitaxel injection 135 mg/m^2^ d1, 3 w + cisplatin 75 mg/m^2^ d1-d3, 3 w).

After two cycles of chemotherapy, reexamination of the enhanced cervical-thoracic CT image showed that the patient was not responding to chemotherapy. On April 20, 2020, he started treatment with the TP regimen (paclitaxel injection 135 mg/m^2^ d1, 3 w + cisplatin 75 mg/m^2^ d1-d3, 3 w) for chemotherapy combined with a PD-1 monoclonal antibody and camrelizumab injections (200 mg/injection).

After the first injection of camrelizumab, the patient did not experience any discomfort and was discharged from the hospital. However, he developed left eyelid ptosis and limited movement of the left eyeball that was accompanied by weakness and soreness in both lower extremities 3 weeks after receiving the first dose of combination treatment. 2 days later, the patient presented with palpitations. Electrocardiogram (ECG) showed sinus tachycardia. Cardiac ultrasound and cardiac function tests revealed the following results: left ventricular ejection fraction 70%, right ventricular ejection fraction 77%, moderate left ventricular diastolic function reduction, and tachycardia. Myocardial enzymatic assays revealed the following results: creatine kinase (CK) 3503.1 U/L (normal value 50-310 U/L), creatine kinase isoenzyme (CK-MB) 178.7 U/L (normal value 0-25.0 U/L), lactate dehydrogenase (LDH) 622.6 U/L (normal value 120-250 U/L), α-hydroxybutyric acid 596.0 U/L (normal value 72-182 U/L) and myoglobin (Myo) 1248 μg/L (normal value 1.5-70 μg/L); cardiac troponin (cTnI) level was 0.35 ng/mL (normal value < 0.040 ng/mL) and rose to 0.63 ng/mL 6 hours later, and brain natriuretic peptide (BNP) is normal. Abnormal liver function was evidenced by the following results: alanine aminotransferase (ALT) 154.3 U/L (normal value 9-50 U/L) and AST 204.4 U/L (normal value 15-40 U/L). On electromyography, the examined muscles showed myogenic damage, and the left deltoid showed a spontaneous spot (active phase) manifestation (**Figure 1C**). Considering the clinical manifestations and laboratory test results, we considered camrelizumab injection-induced adverse myositis involving the myocardium and skeletal muscles accompanied by elevated transaminases, which is considered a grade 4 camrelizumab-induced adverse reaction according to the Common Terminology Criteria for Adverse Events (CTCAE) (The order of occurrence as shown in **Figure 1D**). The patient was immediately injected with methylprednisolone sodium succinate (120 mg daily days 1-5, with gradually decreasing doses); he complained of lower extremity weakness, but his soreness was significantly improved. On the sixth day, the methylprednisolone sodium succinate dose was reduced to 80 mg daily. The patient experienced rapid breathing and dyspnea. Blood gas analysis indicated type II respiratory failure. The patient was transferred to the intensive care unit (ICU), tracheal intubation was immediately performed, and the patient was connected to a ventilator to facilitate respiration. Cardiac troponin (cTnI) level rose to 0.89 ng/mL. Anti-acetylcholine receptor antibody (anti-AChR-Ab) was detected at 10.94 nmol/L. Camrelizumab-associated MG (CTCAE grade 4) was considered. The patient received oral pyridostigmine bromide (30 mg qid with gradually decreasing doses) to improve muscle weakness. Methylprednisolone sodium succinate was restarted at 120 mg with gradually decreasing doses. After five days, the patient’s respiratory condition did not improve. Immunoglobulin injections were administered for one week.

The patient developed ventilator-associated pneumonia during hospitalization in the ICU. Sputum culture results showed *Klebsiella pneumoniae* and pan-drug-resistant *Acinetobacter baumannii*. After two weeks of anti-infective therapy with cefoperazone and sulbactam combined with tigecycline, the patient was weaned off the ventilator and was transferred to the general ward. Within two months, his dose of glucocorticoid therapy was gradually reduced and the levels of biomarkers of myocardial injury declined. His muscle strength gradually recovered, and he returned home to recuperate. During this period, he was treated with oral prednisone tablets (15 mg qd with gradually decreasing doses) and pyridostigmine bromide (30 mg tid).

At a follow-up examination two months later, cervical-thoracic enhanced CT showed a mass in the esophageal wall at the upper thoracic segment, with no obvious change compared to the previous examination, and enlarged lymph nodes on the right supraclavicular fossa and both sides of the tracheal sulcus, with no obvious change compared with the previous film. Cardiac magnetic resonance and ultrasound imaging results were all normal, and myocardial enzymes and liver and kidney functions were normal. Anti-AChR-Ab levels decreased slowly but did not reach a normal level.

## Discussion

Camrelizumab is a humanized high-affinity IgG4 monoclonal antibody against PD-1 (14). It binds to and blocks PD-1 for its binding to the ligand PD-L1 which is overexpressed on activated T lymphocytes, B cells, and natural killer (NK) cells in certain tumors, and PD-L2, which is primarily expressed on antigen-presenting cells. Camrelizumab prevents the activation of PD-1 and its downstream signaling pathways and restores immune function through the activation of cytotoxic T lymphocytes and cell-mediated immune responses directed against tumor cells or pathogens (15). Camrelizumab showed a high dose-dependent affinity for PD-1 (KD 3.31 nmol/L) when administered as a single 60-, 200-, or 400-mg intravenous treatment for patients with solid tumors (10). With a single 200-mg injection of camrelizumab, the peak receptor occupancy of circulating T lymphocytes was 85%, and receptor occupancy remained steadily high in patients who received repeated infusions (once every two weeks), with a receptor occupancy of 77% at the trough concentration after the first infusion of treatment cycle 5. The administration of a single 200-mg IV infusion of camrelizumab to patients with solid tumors (n = 12) produced a mean Cmax of 70.4 µg/mL after a median of 0.00347 days (time to the maximum concentration, tmax) and an area under the curve from zero to infinity (AUC∞) of 465 µg day/mL, and the mean half-life (t½) was 5.61 days (14). The elimination half-lives of PD-1 inhibitors are generally long, and they exhibit slow elimination. When serious adverse drug reactions occur, physicians must cease administration of the drug immediately to avoid drug accumulation and aggravate adverse reactions.

The incidence of immune-associated myocarditis is < 1% (16). ICI-induced myocarditis may be more common than previously thought because of its nonspecific clinical manifestations and the lack of methods for the routine detection of cardiac biomarkers (17). From 2009 to 2018, 613 fatal toxic events caused by ICIs were reported in VigiBase (WHO database). These included 333 deaths related to PD-1/PD-L1 inhibitors, including 27 deaths due to myocarditis (accounting for 8%) and 87 deaths related to the combination of CTLA-4 and PD-1/PD-L1 inhibitors, 22 of them were caused by myocarditis (accounting for 25%) (16). These fatal events indicate that the incidence and mortality of myocarditis significantly increases with combined CTLA-4 and PD-1/PD-L1 inhibitor therapy. The cardiac toxicity of ICIs was diagnosed based on the patients’ drug history, clinical manifestations, cardiac biomarkers, electrocardiogram (ECG) results, endomyocardial biopsy, and imaging examinations. A single center study in China described 283 patients who received PD-1 or PD-L1 inhibitor monotherapy or combination therapy between January 1, 2018, and December 31, 2019: three of the patients had immune-related myositis (incidence: 1.06%), including two patients who received nivolumab monotherapy and one patient who received combination treatment with camrelizumab and gemcitabine, and both patients died (17). In another multicenter, randomized, nonblinded phase III clinical trial, one patient among 228 patients with metastatic esophageal squamous cell carcinoma who received camrelizumab treatment developed myocarditis and died (18). Because most cases of immune-related myocarditis occur at the early stage of ICI treatment and rapidly deteriorate, baseline examination and regular monitoring of myocardial markers and ECG are necessary for the early detection of myocarditis (17).

ICI-induced neuromuscular side effects are rare but often severe and include musculoskeletal pain, myositis, polymyalgia rheumatica, ocular myositis, and MG. Myositis occurs in 1% of patients treated with PD-1 inhibitors (according to the prescribing information for Opdivo^®^) (19); there was one fatality in a trial of pembrolizumab as an adjuvant treatment and a mortality rate less than 1% in patients treated with ipilimumab (according to the prescribing information for Yervoy^®^) (20). A Japanese study of 10,277 patients who received monotherapy with either nivolumab or ipilimumab reported 12 cases of MG among the patients treated with nivolumab but none among the patients treated with ipilimumab, indicating that the incidence of PD-1 inhibitor-induced MG was higher than the incidence of CTLA-4 inhibitor-induced MG (21). Nivolumab-induced myositis (22) and CTLA-4 inhibitor-induced ophthalmic myositis myopathy have been reported (23). These drugs have also been reported to induce severe MG-induced respiratory disorders when used alone or in combination (24).

The side-effect registry and institutional databases of 10 skin cancer centers were queried for reports of myositis and neuromuscular side effects induced by ICIs. Myositis (19/38) was the most frequently reported neuromuscular adverse event. Thirty-two percent of myositis patients had myocarditis (n = 6), 5% of myositis patients had MG, and two patients died from myositis, which is consistent with findings from a WHO registry: myocarditis was associated with myositis in 25% of cases and with MG in 11% of cases. A death case of MG with immune-related myositis involving the myocardium has been reported after camrelizumab treatment (25). Surprisingly, MG also occurred concomitantly in 11 (10%) of 101 patients with myocarditis (26). The specific mechanism underlying myositis associated with myocarditis and MG remains unknown. However, the mechanism might reflect a shared antigen profile and immune phenotype between cardiac and skeletal muscles (17).

In the case of grade 3–4 immune-related myocarditis/myositis (27), ICI treatment must be discontinued, and intravenous glucocorticoids should be started as soon as possible. The initial dosage of prednisone is 1–2 mg/kg daily (or an equal dose of methylprednisolone). If immune-related myocarditis/myositis is accompanied by severe damage (weakness, severely restricted activities, cardiac respiratory insufficiency, difficulty swallowing), plasma exchange and combined intravenous immunoglobulin therapy can also be considered. If symptoms do not improve within two to three days, immunosuppressants should be increased or changed. Recently, two cases of immunotherapy-related myocarditis were reported in the literature. After treatment with alemtuzumab and abatacept, the condition was controlled (6, 7). In this study, the patient received an intravenous injection of a large dose of glucocorticoids combined with gamma immunoglobulin as the main therapeutic drug, with improved results. Glucocorticoid maintenance treatment can take place for four to six weeks after symptoms improve to ≤ grade 1. Patients with myositis respond well to steroid therapy. Notably, doctors should be cautious of recurrent conditions during the dose reduction process of their patients. Shortness of breath and an increased heart rate occurred in this patient during the first dose reduction of methylprednisolone. Blood gas analysis showed that the patient had type II respiratory failure and respiratory acidosis. Progressive aggravation of myositis involving the respiratory muscles was considered. The severity of muscle weakness increased, and the dose of methylprednisolone was increased to and maintained at 120 mg. The dose was reduced after the patient’s condition improved. The case revealed that patients usually can benefit from high dose of glucocorticoid and a slower course of dose reduction.

Supportive care is also very important. The patient was transferred to the ICU for ventilator-assisted ventilation when type II respiratory failure occurred, which led to ventilator-associated pneumonia. Therefore, weaning from the ventilator as early as possible is very important. When a lung infection occurs, the ability to treat the patient with the correct antibiotic will determine the hospitalization time and rehabilitation.

Treatment for MG usually involves symptomatic treatment and immunosuppressive therapy (28). Pyridostigmine bromide, an acetylcholinesterase inhibitor, should be considered as part of the initial treatment for most patients with MG. All MG patients who have received a sufficient amount of pyridostigmine bromide without achieving the treatment goal should receive glucocorticoids or immunosuppressants. This patient was treated with a PD-1 inhibitor and had immune-related myositis involving the myocardium and respiratory muscles accompanied by MG. Therefore, pyridostigmine bromide served as a symptomatic immunotherapeutic treatment.

Lau (29) and Zhu (30) reported that patients with a history of MG showed muscle weakness after receiving pembrolizumab for the treatment of metastatic melanoma. In addition, some patients had high levels of anti-AChR-Ab before receiving nivolumab. After they received treatment, a myasthenic crisis was reported (31). Therefore, for patients with a history of MG, the use of ICIs might activate the T cell autoimmune response and may induce MG.

For patients with a history of MG or other immune-related diseases, physicians should weigh the risk of serious immune-related adverse reactions caused by immune activation due to the use of ICIs. ICI-induced myositis is rare but serious and sometimes fatal. The immediate discontinuation of immunotherapy and timely administration of adequate steroids can reduce the risk of death. Pyridostigmine bromide can be used to treat immune-related polymyositis with MG caused by ICIs and can reduce sequelae. While we emphasize the importance of tumor killing with ICI therapy, we focus on being alert to autoimmune complications in patients who have received ICI therapy, even after the discontinuation of ICI treatment. Early identification and effective management of irAEs are very important.

## Data Availability Statement

The original contributions presented in the study are included in the article/supplementary material. Further inquiries can be directed to the corresponding authors.

## Ethics Statement

Written informed consent was obtained from the individual(s) for the publication of any potentially identifiable images or data included in this article.

## Author Contributions

FZ: Conceptualization, project administration, funding acquisition, writing – review and editing. WD, RF: Writing – review and editing. JL, YW, QL: Supervision. JB: Writing – original draft, formal analysis. KX: Case tracking. PY: Literature review. DL: Data collection. All authors contributed to the article and approved the submitted version.

## Funding

Support was provided by the Hebei Natural Science Foundation (No. H2019206614; Shijiazhuang, China).

## Conflict of Interest

The authors declare that the research was conducted in the absence of any commercial or financial relationships that could be construed as a potential conflict of interest.

## Publisher’s Note

All claims expressed in this article are solely those of the authors and do not necessarily represent those of their affiliated organizations, or those of the publisher, the editors and the reviewers. Any product that may be evaluated in this article, or claim that may be made by its manufacturer, is not guaranteed or endorsed by the publisher.
